# Developing and disseminating an electronic penicillin allergy de-labelling tool using the model for improvement framework

**DOI:** 10.1186/s13223-024-00942-3

**Published:** 2024-12-23

**Authors:** Sujen Saravanabavan, Patrick McKernan, Scott Cameron, Natasha Kwan, Kristopher T. Kang, Ashley Roberts, Roxane Carr, Raymond Mak, Chelsea Elwood, Vanessa Paquette, Rochelle Stimpson, Bethina Abrahams, Edmond S. Chan, Kathryn Slayter, Alicia Rahier, Irina Sainchuk, Sharla Olsen, Melissa Kucey, Jinan Shamseddine, Zahir Osman Eltahir Babiker, Tiffany Wong

**Affiliations:** 1 BC Children’s Hospital, 4480 Oak Street, Room 1C31B, Oak Street Entrance, 1982 Building, Djavid Mowafaghian Wing, Vancouver, BC V6H 3V4 Canada; 2 Faculty of Medicine, University of British Columbia, Vancouver, BC Canada; 3 Division of Allergy, Department of Pediatrics, University of British Columbia, Vancouver, BC Canada; 4 Community Allergy Clinic, Victoria, BC Canada; 5 BC Women’s Hospital and Health Centre, Vancouver, BC Canada; 6 Department of Obstetrics and Gynecology, University of British Columbia, Vancouver, BC Canada; 7 BC Centre for Disease Control, Vancouver, BC Canada; 8 Provincial Health Services Authority, Vancouver, BC Canada; 9 IWK Health Centre, Halifax, Halifax, NS Canada; 10 University Hospital of Northern British Columbia, Prince George, BC Canada; 11 Regina General Hospital, Regina, SK Canada; 12 Department of Pharmacy, Sheikh Shakhbout Medical City, PO Box 11001, Abu Dhabi, United Arab Emirates; 13 Tropical and Infectious Diseases Division, Sheikh Shakhbout Medical City, PO Box 11001, Abu Dhabi, United Arab Emirates; 14 College of Medicine and Health Sciences, Khalifa University, PO Box 127788, Abu Dhabi, United Arab Emirates

**Keywords:** Drug hypersensitivity, Penicillin hypersensitivity, Penicillin allergy, Antibiotic allergy, Patient safety, Quality improvement, Decision support systems, Clinical, Antibiotic allergy

## Abstract

**Background:**

Many clinicians feel uncomfortable with de-labelling penicillin allergies despite ample safety data. Point of care tools effectively support providers with de-labelling. This study’s objective was to increase the number of providers intending to pursue a penicillin oral challenge by 15% by February 2023.

**Methods:**

A validated de-labelling algorithm was translated into an electronic point of care tool and disseminated to eight healthcare institutions. Applying the Model for Improvement Framework, three PDSA cycles were conducted, where collected data and completed surveys were analysed to implement changes. Number of providers intending to pursue an oral challenge, tool usage as well as number of clinicians who felt satisfied with the tool and felt confident in its ability to risk-stratify patients was collected.

**Results:**

50.4% of providers intended to give an oral challenge of penicillin with version 1, which improved to 65.5% with version 2, representing a 15.1% increase. With version 1 of the tool, there was an average of 61.3 counts of tool usage per month. 73.1% of providers felt satisfied with the tool and 76.9% felt confident in its ability to risk-stratify patients. With version 2 of the tool, after implementing changes through three PDSA cycles, monthly usage counts increased to an average of 98.6. Furthermore, 100.0% of providers felt satisfied with the tool and 98.1% felt confident with the tool’s ability to risk-stratify patients.

**Conclusion:**

Our quality improvement approach demonstrated improvement in the percentage of providers that intended to pursue an oral challenge and felt satisfied and confident in the risk-stratification capabilities of penicillin allergy de-labelling tool. Electronic tools should be further incorporated into institutional penicillin de-labelling protocols.

**Supplementary Information:**

The online version contains supplementary material available at 10.1186/s13223-024-00942-3.

## Background

Β-lactams are first line antibiotics for many indications such as common bacterial infections as well as surgical and dental procedure prophylaxis. The high prevalence of inaccurate penicillin allergies is well-defined, with 8–25% of patients globally diagnosed with penicillin allergy despite up to 98% of these patients actually being penicillin tolerant [1–5]. The high rates of penicillin allergy labels has been described as a public health problem resulting in increased rates of antimicrobial resistance, suboptimal treatment of infections and increased adverse events [2, 6, 7].

There is ample evidence documenting various approaches to de-labelling penicillin allergies in inpatient and outpatient settings, including pediatric and obstetric patients [8–11]. Recent literature has supported the implementation of bedside clinical guidelines to support providers with de-labelling penicillin allergies, resulting in a sevenfold increase in β-lactam prescriptions, without increased adverse drug reactions [12, 13]. Notably, computerized penicillin de-labelling guidelines increased penicillin and cephalosporin use twofold [14]. A recent study evaluating a digital antibiotic allergy decision support tool demonstrated an increased proportion of correct clinical decisions in low and medium-risk situations, and appropriate allergist consultation in higher risk situations [15]. Furthermore, ninety-six percent of providers felt that implementation of a digital assessment tool improved their antibiotic selection for patients with antibiotic allergies [15]. An optimal electronic tool is one that is accessible, widely used and user-friendly.

To build on this literature, our centre developed an electronic penicillin allergy risk-assessment tool. The tool was adapted from a study by Roberts et al., which developed and validated the first pediatric electronic algorithm for risk stratifying penicillin allergies [9] and has since been validated in an adult population. This point of care digital assessment tool was created to support providers in risk-stratifying patients with penicillin allergy labels as not allergic, very low risk of allergy, possibly allergic and high risk of allergy and provide recommendations based on their risk category, including the process of de-labelling low risk patients. We used the Model for Improvement framework to refine this tool using an iterative evaluation process guided by survey-based methodology and extracted data from providers using the tool for clinical care [16].

## Methods

### Context

#### Root-cause-analysis

A root-cause-analysis was conducted prior to designing our tool which included a fishbone diagram (Supplementary Fig. 1) and the “Five Whys” technique to identify barriers to de-label penicillin allergies. Multi-disciplinary meetings were held to discuss solutions. The lack of accessible tools to support clinicians with de-labelling penicillin allergies prompted the team to initiate development of an electronic penicillin allergy de-labelling tool.

#### Electronic penicillin allergy de-labelling tool development, validation, and dissemination

Between July 2016 and May 2018, a validation and reliability study was conducted on the first pediatric electronic algorithm, illustrating accurate and safe risk stratification of patients with penicillin allergies [9]. In January 2020, we initiated work with a mobile electronic antimicrobial stewardship platform (Firstline) to host the algorithm through a point of care assessment tool. The development of the electronic algorithm involved stakeholder engagement with infectious disease physicians and pharmacists, pediatricians, allergists, primary care physicians, obstetricians, and antimicrobial stewardship pharmacists. Various mechanisms were put in-place ensure appropriate use of the electronic tool, including educational sessions and dissemination of penicillin allergy de-labelling resources. A beta-test of the tool was distributed from May to June 2021 in adult patients at BC Women’s Hospital (BCWH) in PDSA cycle 1.

#### Settings and target population

This was a multi-center project where providers used the electronic penicillin allergy de-labelling tool in real-world settings. Providers identified and assessed inpatients and outpatients with a penicillin allergy label, including pediatric, adult, and obstetric populations. Version 1 of the tool was distributed to BC Children’s Hospital (BCCH), BCWH, BC Vancouver Island Health (VIHA), which included both adult and pediatric patients. Specific dates of survey and data collection varied for each institution but ranged from June 2021-January 2022. Version 2 of the tool was adopted by BCCH, BCWH, BC Centre for Disease Control (BC CDC), IWK Health (IWK), Northern Health Authority (Northern), Shiekh Shakhbout Medical City (SSMC) and the Saskatchewan Health Authority (SHA). Data collection varied for each institution but ranged from January 2022–February 2023. Any participants outside of these institutions that completed a survey was not included as the survey platform did not record these entries.

#### Oral challenge protocol

The electronic de-labelling tool recommends a single dose challenge. For children, a single amoxicillin dose at 15–30 mg/kg orally (maximum of 500 mg) was suggested. For adults, a single 500 mg tablet of amoxicillin is the recommended challenge dose. 30–60 min of observation in a monitored setting is recommended.

### Interventions

This study used PDSA cycles to implement, assess and improve the electronic penicillin de-labelling tool. Providers were asked to fill out a survey which asked for the following information: whether participants intended to pursue an oral challenge, rating their satisfaction and confidence with the tool’s ability to risk stratify patients, and qualitative feedback to improve the tool. The study only included responses from participating centers.

**Fig. 1:**
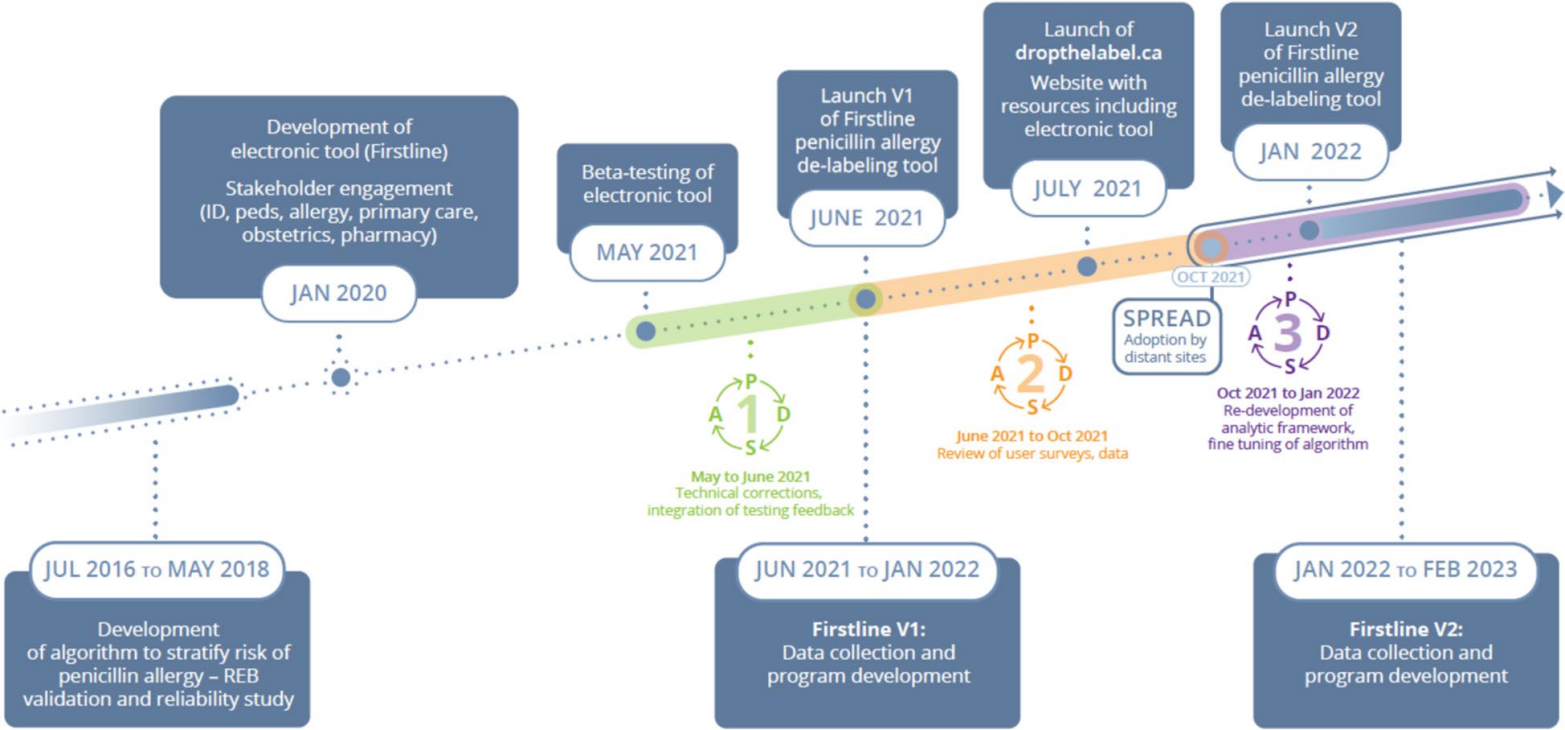
Penicillin electronic de-labelling tool development timeline

We completed three full PDSA cycles (Fig. 1). PDSA cycle 1 (May–June 2021), represented a local beta-test at BCWH, where the objectives were to define and implement technical corrections and integrate testing feedback. Version 1 of the electronic tool was publicly distributed in June 2021. During PDSA cycle 2 (June 2021–October 2021), we acquired survey data, review the data quality and implement changes based on user feedback. To broaden access to the tool, a website www.dropthelabel.ca was released in July 2021 which included penicillin de-labelling resources. In PDSA cycle 3 (October 2021–January 2022), we re-developed the analytic framework and fine-tuned the algorithm based on feedback and data assessment from the previous cycles. Version 2 of the tool was launched in January 2022. Between January 2022–Feburary 2023, we continued to collect data from surveys and focused on program development. Inclusion criteria included any providers that were using the tool to evaluate a patient for clinical purposes. Use of the tool for purposes other than clinical care was excluded from the study.

### Study of the interventions and analysis

Providers were asked whether the tool was for clinical care. If they indicated the data was not for clinical purposes, the data was excluded from the analysis. The tool stratified patients into the following categories: ≥ 18 years old, ≤ 18 years old, or pregnant. We recorded the number of assessments to the following categories: (1) not allergic to penicillin, (2) very low risk for penicillin allergy, (3) possible penicillin allergy, or (4) high risk of penicillin allergy. The data was organized into two sections, “Version 1,” which encompasses data collected during PDSA cycles 1 to 3, and “Version 2,” which was data collected after all three PDSA cycles.

Providers who accessed the electronic tool during the launch and dissemination period were prompted to fill a qualitative feedback survey after use of the tool. The number of providers who intended to proceed with an oral challenge was calculated with each version. Feedback surveys were used to identify key areas of quality improvement. Provider satisfaction and confidence to determine an accurate penicillin risk category (Likert scale 1–5) were calculated within each version of the electronic tool.

Lastly, the following data collected from www.dropthelabel.ca using the website’s analytics function: the total number of page views, the number of unique visits and the number of hits on the website’s resources and de-labelling forms.

### Measures

The outcome measure was the number of providers intending to perform an oral challenge after using the electronic penicillin de-labelling tool. Our process variable was the number of times the electronic tool was accessed, which was collected using website analytics. Balancing measures were provider satisfaction and confidence in the tool’s ability to risk-stratify patients. Qualitative feedback collected during the study phase of each PDSA cycle was applied to the tool to improve the de-labelling framework. Other secondary outcome measures included the number of patients in each penicillin allergy risk category and the demographics of the patients the tool was used for.

### Ethical considerations

A waiver was granted from the BC Children’s Hospital Research Institute due to the quality improvement nature of this project. This project was completed in collaboration with the British Columbia Provincial Health Services Authority Physician Quality Improvement Program. Patients were receiving standard medical appointments and provide consent within this scope—as such a separate consent declaration for participation in this project was not applicable.

## Results

### Participant demographics

Of the 104 participants who completed the feedback surveys, 46 (44.3%) worked in a hospital setting, 56 (53.9%) worked in a community setting and 2 (1.9%) did not indicate work setting. There was an increase in proportion of community vs hospital providers with Version 2 of the tool. In terms of practice location, 93 (89.4%) of the responses came from British Columbia, whereas 11 (10.6%) of the responses came from outside of British Columbia. The demographics of participants is shown in Table 1.

**Table 1:** Practice setting and location of providers who used the electronic penicillin de-labelling tool

	Electronic tool—version 1 (June 2021–January 2022)	Electronic tool—version 2 (January 2022–February 2023)	Total
Practice setting			
Hospital	32	14	46
Community	18	38	56
No entry	2	0	2
Institution (location)			
BC Children’s Hospital (British Columbia)	4	0	4
BC Women’s Hospital (British Columbia)	45	15	60
BC Centre for Disease Control	0	0	0
Island Health (British Columbia)	3	0	3
IWK Health Centre (Halifax)	0	5	5
Northern Health Authority (British Columbia)	0	30	30
Saskatchewan Health Authority (Saskatchewan)	0	2	2
Shiekh Shakhbout Medical City (United Arab Emirates)	0	0	0
Total	52 (50%)	52 (50%)	104

### Outcome measure—intention to perform an oral challenge

With the version 1 (June 2021–January 2022) of the electronic tool, 127 providers used the tool for clinical purposes. Sixty-four (50.4%) providers indicated that they intended to give an oral challenge after using the tool. With version 2 (January 2022–February 2023) of the tool, of the 165 providers that used the tool, 108 (65.5%) indicated they would provide an oral challenge. This data is shown in Table 2. More respondents answered the question about the oral challenge, leading to discrepancy in the number of providers who provided practice demographics, satisfaction scores and confidence scores and the number of providers who intended to give an oral challenge (104 versus 127).

**Table 2:** Intention of providers to do an oral challenge after use of the penicillin de-labelling electronic tool

Intention to do oral challenge	Electronic tool—Version 1 (June 2021–January 2022)	Electronic tool—version 2 (January 2022–February 2023)	Total
Yes	64	108	172
No	63	57	120
Total	127	165	192

Beta testing (May–June 2021): 26 of 38 (68.4%) intended to do an oral challenge

Electronic tool—version 1 (June 2021–January 2022): 64 of 127 (50.4%) intended to do an oral challenge

Electronic tool—version 2 (January 2022–February 2023): 108 of 165 (65.5%) intended to do an oral challenge

### Process measures—usage data

**Fig. 2:**
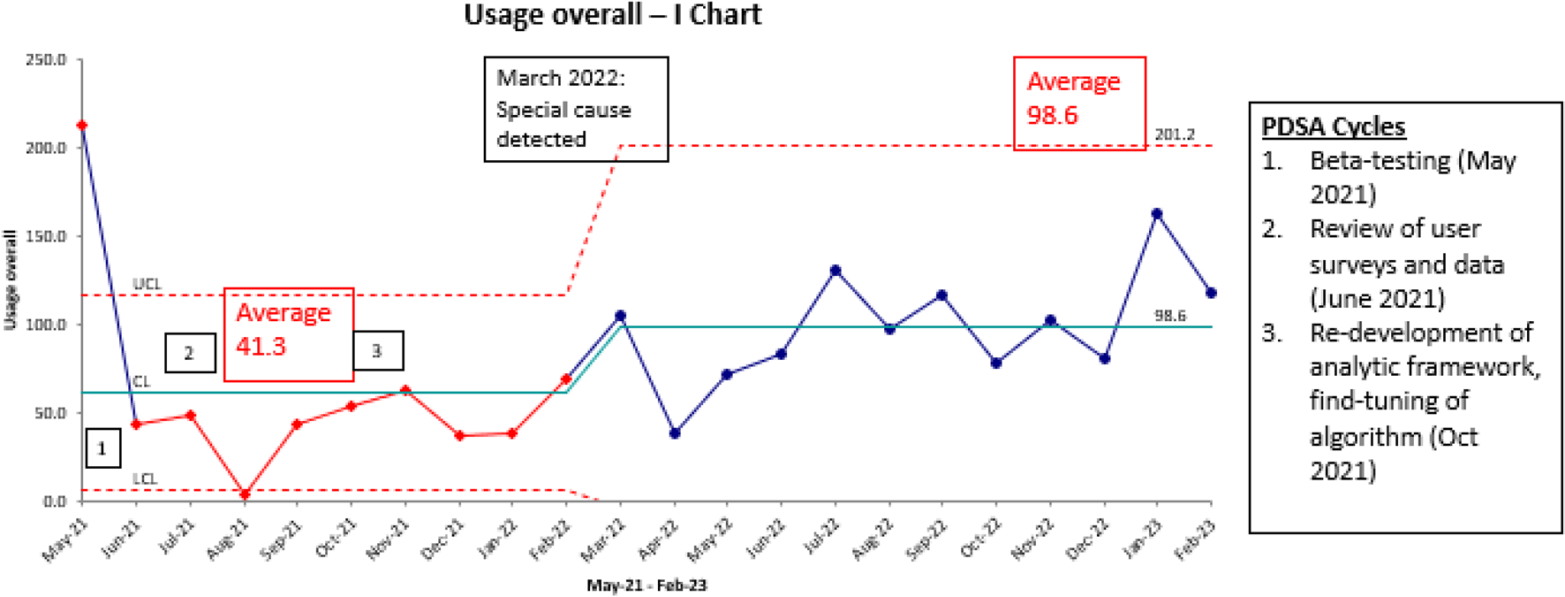
Combined usage of penicillin electronic de-labelling tool across all applications by month from May 2021 to February 2023 Control limits are set at ± 3 sigma units. *LCL* lower control limit, *CL* control limit, *UCL* upper control limit

We assessed usage of the electronic tool across all applications in an I Chart (Fig. 2). There was improvement in usage with average of 41.3 from Jun 2021–Feb 22 increasing to 98.6 from Mar 2022–Feb 2023. There was special cause, which represents a change in data patterns from an intervention or unusual event, detected in March 2022.

### Balancing measures—provider satisfaction and confidence

In total, 86.5% of participants rated their satisfaction with the tool as 4 or 5 out of 5. Furthermore, 87.5% of participants rated their confidence in the decision-making tool as 4 or 5 out of 5. The detailed breakdown of participant satisfaction and confidence is shown in Tables 3 and 4.

**Table 3:** Provider satisfaction with the electronic de-labelling tool

Satisfaction score	Electronic tool—version 1 (June 2021–January 2022)	Electronic tool—version 2 (January 2022–February 2023)	Total
5	31	49	80
4	7	3	10
3	10	0	10
2	2	0	2
1	0	0	0
No entry	2	0	2
Total	52 (50%)	52 (50%)	104

Beta testing (May–June 2021): 76.3% rated it 4 or 5

Electronic tool—version 1 (June 2021–January 2022): 73.1% rated it 4 or 5

Electronic tool—version 2 (January 2022–February 2023): 100.0% rated it 4 or

**Table 4:** Provider confidence in electronic de-labelling tool’s ability to determine risk of penicillin allergy

Confidence Score:	Electronic tool—version 1 (June 2021–January 2022)	Electronic tool—version 2 (January 2022–February 2023)	Total
5	30	49	79
4	10	2	12
3	4	0	4
2	4	0	4
1	2	1	3
No entry	2	0	2
Total:	52 (50%)	52 (50%)	104

Beta testing (May to June 2021): 76.3% rated it 4 or 5

Electronic tool—version 1 (June 2021–Jan 2022): 76.9% rated it 4 or 5

Electronic tool—version 2 (January 2022–February 2023): 98.1% rated it 4 or 5

### Patient risk category

In total, 1,432 patients had their penicillin allergy assessed. With version 1 (June 2021–January 2022) of the electronic tool, 642 patients were reported to be assessed. Eighty-nine (13.7%) patients were deemed not allergic, 371 (57.8%) had a very low risk of allergy, 114 (17.6%) had a possible allergy and 68 (10.6%) were deemed to be high risk of a beta-lactam allergy. With version 2 (January 2022–February 2023), of the 790 patients assessed for clinical purposes, 234 (29.6%) patients were not allergic, 317 (49.4%) had a very low risk of allergy, 119 (15.1%) had a possible allergy and 40 (5.1%) were found to be high risk.

### Survey feedback

In terms of the feedback surveys, a common challenge for providers was the inability to complete the algorithm due to incomplete details of the reaction on history. There was also more minor feedback regarding wording of various questions and incorporation of data within the tool. Other comments expressed that the tool was useful and time efficient. Details of the changes made with each version of the tool is shown in Table 5 in the supplementary data. Examples of various survey results are included in Table 6 in the supplementary data.

## Discussion

This penicillin allergy electronic de-labelling tool was assessed in a multi-center Canadian context, including implementation at a site in the United Arab Emirates, with providers in hospital and community settings. The tool’s efficacy was inferred by the number of providers who intended to pursue an oral challenge to patients based on their risk category. 50.4% of participants indicated they wanted to perform oral challenges with the first version of the tool, which increased to 65.5% with the second version of the tool, representing a 15.1% increase, achieving the overall aim of this project. Although there was an increase in the number of providers intending to pursue an oral challenge, this rate is likely much lower than actual number oral challenges given. This likely reflects the tool’s structure and many respondents exiting the tool before completing the remainder of the algorithm’s questions after determining the patient’s risk-category. We also demonstrated that there was an increase in provider satisfaction and confidence in the tool and its ability to determine the risk of a penicillin allergy. We also showed an increase in uptake and usage of the electronic tool over time, illustrated by the increased number of community providers using version 2 of the tool compared with version 1. Regarding the special cause identified in March 2022, the increased uptake may be attributed to growing awareness of this electronic resource, as well as improvements with the application. There were also parallel initiatives which may have contributed, including various teaching initiatives such as webinars, continuing medical education and resident teaching. With version 1 of the tool, 10.6% (68/642) of patients assessed with this tool were found to be high risk of having a penicillin allergy. Some of these “patients” are likely to have come from providers who were testing the tool for non-clinical purposes, as the survey question asking if the provider was using the tool for clinical purposes was placed at the end of assessment. With version 2, the question was introduced of whether provider was using the tool for clinical purposes was placed prior to assessment. We then found that 5.1% (40/790) of patients were found to be high risk of having a penicillin allergy, which is consistent with current epidemiologic data.

These findings are consistent with a study by Dunham et al., where a digital support tool for beta-lactam allergy management was tested by non-allergist providers to make antibiotic de-labelling decisions in test cases, with and without the tool. Their validated algorithm was also accessible through all electronic devices or webpage. Similar to our study, they demonstrated a high degree of confidence with the de-labelling tool with participant confidence from 17.6% participants feeling confident without the tool to 80.0% feeling confident with the tool [15].

A limitation in this study is that with version 1 of the tool, the question asking participants if they were using this tool for clinical purposes came at the end of the survey. Therefore, some of the results with the first version of the electronic tool may have been from participants testing the tool. These issues were mitigated with the second version of the tool, where this question was moved to the beginning of the tool, allowing us to exclude anyone testing the tool from our study. Furthermore, a large proportion of our participants were from British Columbia, which may impact the study’s generalizability to other populations. Another limitation of this project was the requirement for mobile capabilities to conduct the assessment. While an effort to make the tool widely accessible was made, there will still be practitioners working in remote areas where internet access is unavailable. Additionally, if there is no history available at all, this de-labelling tool cannot be used to conduct an assessment. Given the widespread use of this tool, we were only able to use intention to conduct test dosing as a surrogate for actual challenge. For centers involved academically, we have ongoing data collection to determine whether challenge has been completed and successful. Additionally, barriers to giving oral challenges was not explored. This study also did not assess the tool’s impact on frequency of appropriate penicillin prescriptions. Lastly, safety data wasnot collected, so adverse reactions with oral challenges is unknown, but based on several published studies, we expect oral challenge is safe in appropriately selected low-risk patients [8, 17, 18].

Our electronic tool is freely accessible online (www.dropthelabel.ca) in order to remove barriers to access, as long as the provider has an internet connection. We believe that the high uptake of the electronic tool is related to accessibility. We encourage ongoing development of strategies to disseminate knowledge on the importance of penicillin de-labelling and increase accessibility of available electronic de-labelling tools.

## Conclusion

We used the Model for Improvement to develop an electronic point of care de-labelling tool in a multi-center setting [16]. Implemented changes resulted in an increased intention to pursue an oral challenge and tool usage with a high degree of satisfaction and confidence in the tool’s capabilities to determine risk of penicillin allergies amongst providers. Future studies should examine barriers to implementing electronic de-labelling tools and adhering to its recommendations as well as explore strategies to sustain penicillin allergy de-labelling in diverse practice settings. Further research on how to successfully provide knowledge translation spread and sustain penicillin allergy de-labelling in various practice settings is needed.

## Supplementary Information

Supplementary Material 1.

## Data Availability

Data is provided within the manuscript or supplementary information files.

## Abbreviations

PDSA: Plan-Do-Study-Act
BCWH: BC Women’s Hospital
BCCH: BC Children’s Hospital
VIHA: Island Health
BC CDC: BC Centre for Disease Control
IWK: IWK Health
Northern: Northern Health Authority
SSMC: Shiekh Shakhbout Medical City
SHA: The Saskatchewan Health Authority
