# Supplementary material for: Developing and disseminating an electronic penicillin allergy de-labelling tool using the model for improvement framework
Source: Allergy Asthma Clin Immunol. 2024 Dec 23;20:73. doi: 10.1186/s13223-024-00942-3 (PMC11668003; doi:10.1186/s13223-024-00942-3)
Supplement: Supplementary file 1 — Supplementary Material 1. [file 13223_2024_942_MOESM1_ESM.docx]

**SUPPLEMENTARY DATA:**

**
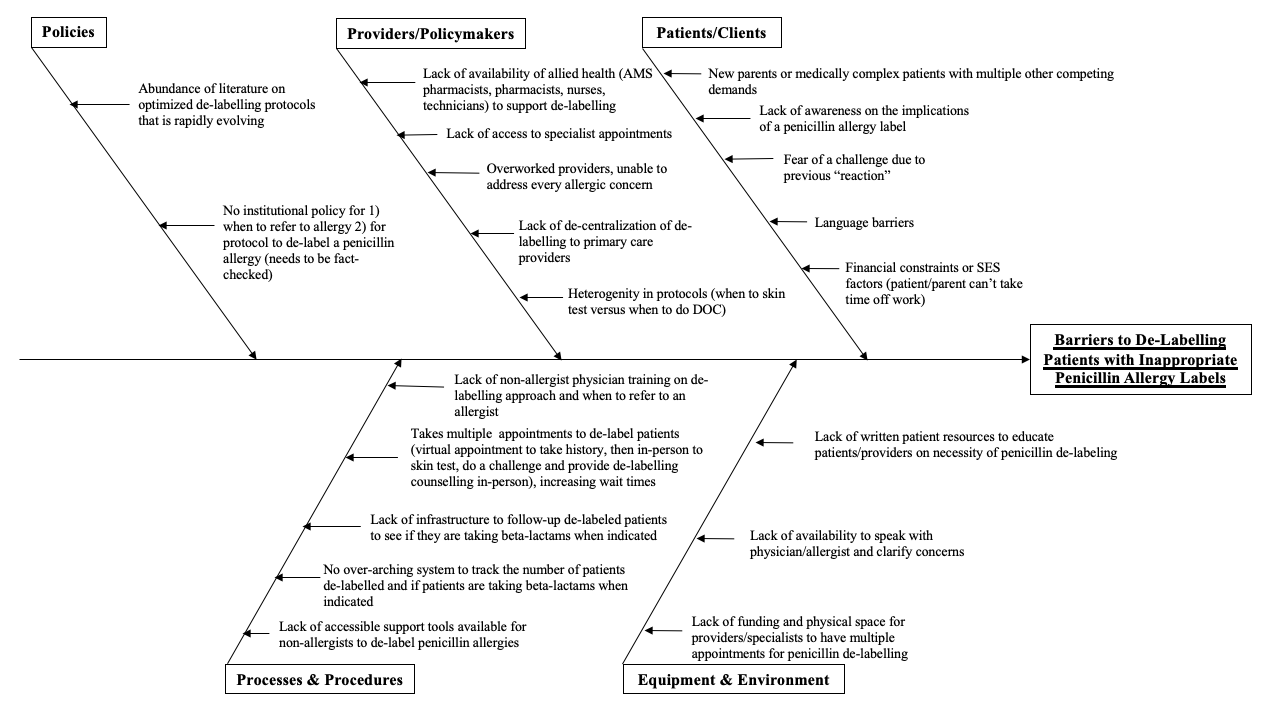
**

**Supplementary Figure 1.** Root-cause-analysis fishbone diagram to illustrate barriers to de-labelling patients with inappropriate penicillin allergy labels

**Table 5.** Summary of changes made to electronic de-labelling tool after each PDSA cycle

|  | PDSA Cycle 1 | PDSA Cycle 2 | PDSA Cycle 3 |
| --- | --- | --- | --- |
| Summary of changes after PDSA cycle | - Grammatical and technical changes - Re-phrasing of questions for clarity of understanding | - Included question asking if user was using the tool for clinical purposes - Improvements to data analytics - Updated algorithm to address patients who did not re-call their history - Grammatical corrections - Further technical changes | - Minor technical corrections - Minor language/grammar revisions |

**Table 6.** Samples of qualitative survey results on the penicillin de-labelling electronic tool

|  | Positive Feedback: | Constructive Feedback: |
| --- | --- | --- |
| Electronic Tool Feedback – Version 1: | Great tool | No option for 'don't recall details' and >10 years ago but not isolated mild cutaneous or recalls severe illness/hospitalization - I assume possible allergy, but would be nice to know |
|  | Amazing tool | This doesn’t help reduce referrals for my common patient who « had rash all over from penicillin as child so never took again » which are majority of what I hear as family doctor ! |
|  | Excellent tool |  |
|  | Really useful tool |  |
| Electronic Tool Feedback – Version 2: | excellent, time effective | The question about allergy/family is a double negative and makes me pause every time! |
|  | Excellent | Unable to fully complete tool - patient doesn't recall details of reaction >10 y ago, was already in hospital for infection being treated, so unclear whether allergic symptoms required hospitalization or had severe symptoms. Doesn't fit under either 'don't recall details' category. |
|  |  | i feel the tool is hard to navigate (no back button). i would also like risk estimates of probabilities. and links to data that support the tool, esp as the tool is not endorsed by any guideline i am aware of. |

Examples of positive and constructive qualitative feedback from surveys for the electronic penicillin allergy de-labelling tool
